# On Assuring Healthy Dentine over Endangered Pulps

**Published:** 1885-02

**Authors:** Jacob L. Williams

**Affiliations:** Boston, Mass


### On Assuring Healthy Dentine Over Endangered Pulps.

BY JACOB L. WILLIAMS, M.D., BOSTON, MASS.

Read in the Section on Oral and Dental Surgery of American Medical Association. May, 1884.

In saying endangered pulps I mean cases where the pulps have but little of the natural covering of dentine in the depths of cavities, which covering is very apt to be more or less tainted by disease, that is by the agents that have produced caries.

Let us look at the conditions here existing. There is acid with fermentive action, often in full sway, seeming at first sight to demand the most decided and effective neutralizing and corrective applications. But just beyond lie some of the most highly and delicately organized tissues of the whole body; fibres, that if rudely set vibrating will be likely to continue or repeat their vibrations in a very serious way; tissue, which if inflamed, there is none more doubtful of survival; and we must remember that the life of the pulp means additional years of usefulness to its organ.

These circumstances I fear are often too little considered in the use of violent or caustic applications.

But what should be done? Perhaps my ideas on this point may be best given by mentioning a plan of treatment for such cases that I formed, tested, and adopted during the years 1847 and 1848; and which in its general principles I have found to be most satisfactory up to the present time. Of course, since that date new materials have been added to our list of available correctives for diseased conditions.

After first removing the loosest debris, I applied a mild antacid such as aqua calcis or a solution of bicarbonate of soda, after thorough saturation with which, I treated the cavity for the correction of fermentive elements by the application of a solution of chloride of calcium, also very dilute creosote (we now have several substitutes for that). The cavity was then dried and sealed up for from two to three weeks, when it was opened and the *mild* saturation repeated, adding perhaps a mild astringent, such as tannic acid in weak solution, to the latter application. Then it was sealed up again, generally for a longer period, and the dressing was repeated at proper intervals until permanent health of dentine was assured.

Now with these mild correctives we have a greater chance of avoiding undue irritation of the pulp than with stronger applications; but they will be more or less transient in their effect, and therefore will require frequent repetition, which may be less frequent as the dentine gains in soundness and health.

By this method we help nature in the line of her preference, that is to protect rather than injure a vital point, keeping the disease in check to give her opportunity.

It may be, and has sometimes been said, that this treatment is very troublesome, and why not make one strong application, and trust that nature will do the rest? I reply that the great objection is that while a strong or caustic dressing is not always lasting in corrective influence, it adds vastly to the possibilities, if it does not make sure, that the reparative action of the pulp will be interfered with, resulting in failure of the ultimate object of secondary deposit. Instances of this sort are often seen in practice; and in my observation the proportion of success of the mild repetition plan is far beyond that of the single capping practice.

Of course there will be some chance of non-success in many attempts to save the pulp, depending often on constitutional as well as local causes, and both may be obscure. The best and most promising efforts may be thwarted by neglect of the patient to report at proper times, or by interruption of the general health. Or, again, when cases are nearly ready for a permanent operation, they may be piratically appropriated by some person who very probably will speak lightly of the treatment that has brought the teeth into condition for his profitable seizure. But still our highest duty is to save life rather than to destroy or endanger it.

Representative cases might be related from a large number with various phases, but to describe them minutely would task both your time and patience.

Plaster of Paris was first used as a cap for the exposed portion. Oxide of tin was also mixed with the gutta-percha.

One rather extreme case I might mention, occurring in 1854.

A young man aged twenty-five, upper left first molar having a large and deep cavity on the mesial side, with the pulp covered only very thinly by softened and disintegrated remains of dentine.

But the pulp was apparently uninjured, and on inquiry I found there had been no pain, and nothing to indicate the existence of inflammation in any degree, which was an encouragement to give the pulp every chance to live and protect itself by secondary dentine.

I saturated the cavity first with aqua calcis about half strength, then with solution of chloride calcium; but this causing slight sensation I reapplied the aqua calcis with the effect of stopping pain.

I then applied a *mild* non-irritating antiseptic in proportion about as follows, viz.: a wood creosote (at that time the best known antiseptic of its kind), one drop to one drachm of alcohol, adding two drachms of water, after which I dried the cavity and covered the depth over the pulp with a mixture of oxide of zinc, five parts to one of gutta percha mixed with its bulk of yellow wax, over which I placed carefully, without pressure, and filling up the cavity with the gutta percha, an oxide of zinc mixture, melting the wax. After two months I unsealed the cavity, repeated the antiseptic with the addition of a trace of tannic acid; this at first caused sensation which was readily dispelled by aqua calcis. I then dried and sealed up the cavity; at the end of four months repeating the above treatment. twice again after six months, and finally after about eight months, making more than two years in all, when there was a firm, hard almost transparent floor of new dentine, from which the former disintegrated material was readily brushed off by a light touch of an instrument. After placing an inert non-conducting material (a piece of fine white silk saturated with wax) at the depth of the cavity, I filled with gold. Two years afterwards a small cavity occurred on the distal side of the same tooth, which on preparing to fill I found as sensitive to the instrument as if it had been the first cavity the tooth ever had, proving the unimpaired vitality of the organ, which continued several years after and during the life of the patient.

DISCUSSION.

Dr. W. W. Allport, Chicago, Ill.—Was certainly very much interested in the paper, and the line of treatment mapped out by the author. It has been the usual practice to apply antiseptics in these cases, creosote, carbolic and eucalyptus, etc., in various strengths, and then to immediately cap with some non-conducting material like gutta-percha, oxychloride, or oxyphosphate of zinc.

The first important point to be gained is to neutralize or destroy the agents of decay, and then to protect the pulp from irritation of every variety.

This can best be done by using a non-conducting material directly over the pulp and then to fill the balance of the cavity with a substance sufficiently hard and firm to prevent irritation from mechanical pressure, but do not see why the treatment need be continued over so long a period as that practiced by Dr. Williams.

Dr. J. L. Williams.—The object in continuing the treatment over so many months is to be sure that the active causes of decay are destroyed or rendered inert, and to guard against pulp irritation by frequent dressing with mild remedies, rather than to run the risk incident to the application of such intense medicaments as are commonly used.

Dr. Allport.—I have heard something recently about a new remedy for the treatment of this class of diseases made from the oil of cloves, and known as eugenol. It has been very highly recommended as having a decided fatal influence over germ life.

Have had no experience with it myself, but would like to hear a report upon the subject from those gentlemen who have experimented with it.

Dr. A. W. Harlan, Chicago, Ill.—Dr. Williams spoke of his treatment being to antagonize the acid or acid ferment. Such treatment could be only of temporary benefit; the remedies must be of sufficient strength to destroy the agents of fermentation. Most practitioners are in the habit of swabbing the cavity of decay with creosote, carbolic acid, and other like remedies, and after temporarily sealing it up for a few days, capping with gutta-percha solution, oxychloride of zinc, metal caps with various forms of dressing placed upon the under surface and permanently filling the cavity of decay. But in such cases we have no means of knowing whether there has really been any deposit of secondary dentine, and I believe in a vast majority of them no secondary deposit occurs.

